# Domain-specific physical activity and affective wellbeing among adolescents: an observational study of the moderating roles of autonomous and controlled motivation

**DOI:** 10.1186/s12966-018-0722-0

**Published:** 2018-09-10

**Authors:** Rhiannon Lee White, Philip D. Parker, David R. Lubans, Freya MacMillan, Rebecca Olson, Thomas Astell-Burt, Chris Lonsdale

**Affiliations:** 1 School of Science and Health, Western Sydney University, Locked Bag 1797, Penrith, NSW 2751 Australia; 2 Institute for Positive Psychology and Education, Australian Catholic University, PO Box 968, North Sydney, NSW 2059 Australia; 3 Priority Research Centre for Physical Activity and Nutrition, University of Newcastle, University Drive, Callaghan, NSW 2308 Australia; 4 School of Social Science, The University of Queensland, QLD, St. Lucia, 4072 Australia; 5 Population Wellbeing and Environment Research Lab (PowerLab), School of Health and Society, University of Wollongong, Northfields Avenue, Wollongong, NSW 2522 Australia; 6 Menzies Centre for Health Policy, University of Sydney, Camperdown, NSW 2006 Australia; 7 School of Public Health, Peking Union Medical College, Tsinghua University and Chinese Academy of Medical Sciences, Beijing, 100006 China

**Keywords:** Physical activity, Exercise, Mental health, Adolescents, Life domain, Motivation, Self-determination theory

## Abstract

**Background:**

Abundant evidence demonstrates a relationship between physical activity and mental wellbeing. However, the strength of the relationship is not consistent. Factors contributing to variation in the strength of association are not well understood and, therefore, it remains difficult to optimize physical activity to ensure the strongest possible relationship with mental health. Self-determination theory suggests that more autonomously motivated behaviors lead to better mental health outcomes, when compared to more controlled behaviors. Therefore, we examined whether autonomous and controlled motivation moderated the relationships between physical activity and affective wellbeing within two domains (i.e., leisure-time and active travel).

**Methods:**

Between February and April 2014, adolescents (*N* = 1632, *M* age = 12.94 years, *SD* = 0.54, 55% male) wore an accelerometer across seven-days and completed self-report measures of leisure-time physical activity and active travel. They also completed two measures of motivation (towards leisure-time physical activity and active travel) and an affective wellbeing measure.

**Results:**

Structural equation modeling revealed that greater self-reported leisure-time physical activity was associated with greater positive affect (β = .29) and less negative affect (β = −.19) and that motivation did not moderate these relationships. Self-reported active travel had no linear relationship with affective wellbeing, and motivation did not moderate these relationships. Accelerometer-measured leisure-time physical activity had no relationship with positive affect but, had a weak inverse association with negative affect (β = −.09), and neither relationship was moderated by motivation. Accelerometer-measured active travel had no association with positive affect; however, autonomous motivation significantly moderated this association such that active travel had a positive association with positive affect when autonomous motivation was high (β = .09), but a negative association when autonomous motivation was low (β = −.07). Accelerometer-measured active travel had no association with negative affect. Despite some significant moderation effects, motivation did not consistently moderate the relationship between all physical activity variables (leisure-time and active travel, and self-report and accelerometer) and affective outcomes.

**Conclusions:**

Tailoring physical activity interventions and guidelines to prioritize leisure-time ahead of other life domains could benefit wellbeing. Promoting autonomous participation in active travel may also be associated with increased wellbeing among adolescents.

**Electronic supplementary material:**

The online version of this article (10.1186/s12966-018-0722-0) contains supplementary material, which is available to authorized users.

## Background

Abundant evidence shows that adolescents who are more physically active experience greater mental wellbeing than their less active counterparts [1–3]. Yet, adolescents accumulate their weekly physical activity (PA) across a number of different life domains (e.g., at school, during leisure-time, and when actively travelling to and from school) [4–6]. A recent meta-analysis showed that the relationship between PA and mental wellbeing is not consistent across these domains, with leisure-time PA having a stronger positive relationship with mental wellbeing, and a stronger inverse association with mental ill-being, when compared to all other domains [7]. Specifically among adolescents, leisure-time PA appears to be an optimal domain for achieving mental health benefits [8–12]. For example, Valois et al. showed that adolescents participating in higher amounts of leisure-time PA experienced reduced odds of life dissatisfaction [13] and McKercher et al. found an inverse relationship between leisure-time PA and depressive symptoms [14]. Conversely, evidence concerning PA at school and on the way to school is limited, and contradictory. While some studies have shown that actively commuting to school and participating in physical education are associated with reduced odds of depression [14, 15], Valois et al. showed that transport PA and physical education had no association with life dissatisfaction [13] and McKercher et al. found no relationship between active travel and depressive symptoms [14]. The conflicting evidence as to whether PA outside leisure-time is beneficial is particularly concerning given recent emphasis has been placed on promoting incidental PA behaviors, as opposed to organized sport during leisure-time [16]. At present, it is unknown whether this approach will be beneficial to mental health and wellbeing.

In an attempt to explain why active travel appears beneficial for some and detrimental for others, Asztalos et al. suggested that individuals who own a car but choose to walk or cycle, and are thus more autonomously motivated, may psychologically benefit from PA, while individuals who actively travel for controlled reasons (e.g., they have no choice because they cannot afford a car) do not experience psychological benefits [17]. This notion may also hold true for adolescents; for example, students who walk or cycle to school because they enjoy it may experience more positive psychological outcomes than those who are forced to actively travel to school by their parents. Indeed, self-determined motivation has been positively associated with positive affective outcomes in physical education [18, 19] and elite sport contexts [20]. These studies support a relationship between motivation and wellbeing in PA contexts but did not examine the relationship between PA and wellbeing. Nevertheless, the potential moderating role of motivation is supported by self-determination theory, which posits that the degree to which a behavior is autonomously motivated influences the effect of that behavior on mental wellbeing, such that, more autonomous behaviors provide greater benefits to mental wellbeing, and controlled behaviors undermine wellbeing [21]. Autonomously motivated behaviors are those in which individuals act with volition and choice because they find the activity enjoyable or personally important [22]. Conversely, controlled motivation refers to engaging in an activity due to internal pressure such as guilt, or external incentives such as rewards or enforcement [23].

Evidence consistently shows that adolescents with higher autonomous motivation towards PA, are more active [24], and therefore, individuals may experience increased mental health benefits from the amount of autonomously motivated PA they undertake. However, if we expect motivation to only predict the amount of activity one engages in, then those who are autonomously motivated would engage in PA and experience mental health benefits, and those who only have controlled motivation would not engage in PA and have poorer wellbeing as a result. However, the large heterogeneity in the strength and direction of the relationship between PA and mental health [7], suggests that not all PA participation is always beneficial to mental health, and indeed some PA experiences may be detrimental to wellbeing. As individuals engage in PA for a variety of reasons, it is possible that those with more autonomous – or more self-determined – motivation, derive more mental health benefits from their participation in PA, while those who participate for more controlled reasons – or have less self-determined motivation – do to a lesser extent. Motivation for active travel to school, in particular, is likely to be more controlled than other domains because most young people have little choice over their mode of travel to school [25]. In these scenarios – where motivation is controlled – it is unknown if adolescents are likely to experience positive mental health benefits due to the PA they have undertaken, or if as self-determination theory suggests, their controlled motivation will undermine wellbeing and lead to negative outcomes. Although leisure-time may inherently provide individuals with more choice over how they spend their time, the degree to which individuals are autonomously motivated may still vary, and many adolescents report both autonomous and controlled motivation towards leisure-time PA [26]. Given that there is still large heterogeneity in the strength of association between leisure-time PA and mental health outcomes among adolescents, it is likely that motivation plays a role in leisure-time also, although perhaps a smaller role than for domains that are obligatory in nature.

Understanding why PA during specific domains improves wellbeing for some individuals and not others is an important endeavor. However, to date, no study has employed a self-determination theory framework to examine if motivation towards specific PA domains contributes to some of the variation in the strength of the relationship between PA and mental wellbeing. If motivation does explain why PA is associated with enhanced wellbeing for some individuals and not others, then PA experiences could be optimized to strengthen the likelihood of experiencing positive mental health outcomes. As such, the primary purpose of this study was to examine whether autonomous and controlled motivation influenced the strength or direction of (i.e., moderated) the relationships between domain-specific PA (i.e., leisure-time PA and active travel) and the affective component of wellbeing among adolescents. To address this aim we developed the following research questions:

1. What is the relationship between leisure-time PA, and positive and negative affect among adolescents?
2. What is the relationship between active travel, and positive and negative affect among adolescents?
3. Are the relationships between leisure-time PA and affect stronger than the relationships between active travel and affect?
4. Do autonomous and/or controlled motivation towards PA moderate the relationships between domain-specific physical activity (i.e., leisure-time and active travel) and positive and negative affect among adolescents?

We hypothesized that overall, leisure-time PA would have a stronger positive association with positive affect, and a stronger inverse relationship with negative affect, compared to active travel. We also hypothesized that the relationships between leisure-time PA and affect, and between active travel and affect, would be moderated by motivation. Specifically, there would be stronger positive relationships between PA and positive affect, when motivation is more autonomous, and stronger positive relationships between PA and negative affect when motivation is more controlled.

## Methods

### Participants

To detect a relationship between PA and affective wellbeing, similar in magnitude to previously reported [17, 27], we required 315 participants for 80% power. To examine the moderating role of motivation, the required sample size needed to be multiplied by four [28], for a final target sample of 1260. We collected data from students in 14 government-funded high schools in Western Sydney, Australia, in 2014 [29]. All schools were located in postcodes with a low socioeconomic status. We invited all Year 8 students without an injury or medical issue preventing their participation in PA. Of the 1806 Year 8 students enrolled, 1632 students provided consent. University and NSW Department of Education ethics committees provided approval.

### Measures

#### Leisure-time PA

We defined leisure-time PA as PA accumulated outside school hours, excluding travel to and from school. Students completed an adapted version of the WHO Health Behavior in School-aged Children measure of PA to self-report their leisure-time PA. Acceptable validity has been reported for Health Behavior in School-aged Children PA scores in a sample of Year 8 Australian students [30]. Given recall errors and social desirability can introduce bias into self-report data, we also used accelerometers (ActiGraph, LLC, Fort Walton Beach, FL) to measure student MVPA during leisure-time. To determine the school day period, we recorded each school’s bell times. We then filtered out both the school day, and the self-reported travel period from the accelerometer data to objectively calculate MVPA during leisure-time only.

#### Active travel PA

Each student systematically completed a 48-h recall travel diary (see Additional file 1: Appendix A) during class. The diary included their mode of travel to and from school, which we used as a self-report measure of active travel, and the time students left home and arrived at school in the morning, and left school and arrived at their first destination in the afternoon [31]. We also used accelerometers (ActiGraph, LLC, Fort Walton Beach, FL) to measure student MVPA during periods of travel to and from school. The accelerometer data was directly derived from each student’s individually self-reported recall travel times recorded in their diary. See details in the procedures section.

#### Motivation towards leisure-time PA

We used the Behavioral Regulation in Exercise Questionnaire (BREQ-2) to measure motivation towards leisure-time PA [32]. The BREQ-2 consisted of the stem “Why do you participate in sport and/or physical activity during your spare time?” The questionnaire then comprises 19 items relating to five motivational regulations: intrinsic motivation (4 items; e.g., I exercise because it’s fun), identified regulation (4 items; e.g., I value the benefits of exercise), introjected regulation (3 items; e.g., I feel guilty when I don’t exercise), external regulation (4 items; e.g., I exercise because other people say I should), and amotivation (4 items; e.g., I don’t see the point in exercising). Participants responded to items using a 5-point Likert scale (*not true for me* = 1 to *very true for me* = 5). Acceptable validity has been shown among adolescents for the 5-factor BREQ-2 [32, 33]. In the present study, internal consistency values ranged from α = .72 to α = .84.

#### Motivation towards active travel

To measure motivation towards active travel to and from school, we developed the Motivation towards Active Travel to School Scale (MATSS). The MATSS included the stem “I actively travel to or from school...” and was comprised of nine items designed to measure autonomous motivation (3 items; e.g., because I enjoy it), controlled motivation (3 items; e.g., because other people (e.g., parents, friends) tell me I should), and amotivation (3 items; e.g., but I feel it is a waste of time). Participants responded to each item on a 5-point Likert scale (*strongly disagree* = 1 to *strongly agree* = 5). In the current sample, internal consistency scores were α = .76 for autonomous motivation, α = .65 for controlled motivation, and α = .79 for amotivation. Full information regarding the development and psychometric testing of the MATSS scores is included in Additional file 2: Appendix B.

#### Affective wellbeing

Affective wellbeing refers to people’s experiences of positive and negative affect [34], and is central to mental wellbeing [35, 36]. We used the short version of the Positive and Negative Affect Schedule for Children (PANAS-C) [37] to measure positive and negative affect. The PANAS-C included the instruction “indicate to what extent you have felt this way during the past few weeks” and was followed by 10 items; five designed to measure positive affect (e.g., happy) and five intended to measure negative affect (e.g., sad). Participants responded to each item on a 5-point Likert scale (*very slightly* = 1 to *extremely* = 5). Evidence supports the validity of this measure in adolescents [37]. Internal consistency of the PANAS-C measurement was also acceptable in the current sample; α = .84 for positive affect and α = .79 for negative affect.

#### Confounders

We measured participants’ height to the nearest 0.1 cm using a portable stadiometer, and weight to the nearest 0.1 kg using digital scales, and calculated each participant’s body mass index (BMI). Participants also completed the Family Affluence Scale as a measure of socioeconomic status [38].

### Procedures

Students completed the motivation questionnaires and the leisure-time PA questionnaire during one scheduled classroom lesson. Research assistants then demonstrated the correct positioning of an accelerometer and requested that students wear the device for the following seven-days. Students received a text message reminder each morning to encourage them to wear the device. During this period, participants also completed the 48-h travel diary during two scheduled lessons 2 days apart.

To prepare the accelerometer data, we created wear-time files consisting of a count-value for each 1-s epoch of data, excluding periods of ≥60 min of consecutive zero counts, allowing for a 1–2 min spike tolerance of 0–100 counts per minute [39]. We then used ActiLife software (Actigraph, LLC, Fort Walton Beach, FL) to specify the time periods to be analyzed for each participant (i.e., leisure-time and active travel). The active travel data period was defined by each student’s self-reported recall travel times, and the leisure-time data consisted of all wear-time counts, minus the school day (defined by school bells) and the active travel period. Within each domain, we categorized each epoch count-value based on the intensity of raw acceleration into its corresponding intensity of PA (i.e., sedentary, light, moderate, or vigorous) based on Evenson and colleagues’ equations [40].

### Statistical analysis

First, we calculated Pearson product-moment correlations to examine the relationships between all variables (Table 1). Next, to answer Research Questions 1 and 2, we conducted structural equation modelling in MPlus (version 7.4) [41] to test the hypothesized relationships between domain-specific PA (independent variables) and the two components of affective wellbeing (dependent variables). We used full information maximum likelihood estimation to account for the 9.53% of data points missing and employed robust standard errors (MLR) to handle potential violations of multivariate normality and the complex nature of the data (students nested within schools). Second, to answer Research Question 3, we constrained corresponding paths (e.g., leisure-time PA on positive affect and active travel on positive affect) to be equal and conducted a Wald test to determine if there was a significant difference between the two paths.

**Table 1:** Correlations between Physical Activity, Affect, and Motivation

Variable	*N*	M	SD	Positive Affect	Negative Affect	Leisure-time PA self-report	Active Travel self-report	Leisure-time PA objective	Active Travel objective	BREQ-2 autonomous	BREQ −2 controlled	MATSS autonomous	MATSS controlled
Positive Affect	1403	3.64	0.89										
Negative Affect	1403	1.89	0.84	−.33***									
Leisure-time PA: self-report	1317	3.41	1.20	.25***	−.15***								
Active Travel: self-report	1414	0.50	0.43	−.04	−.00	.06							
Leisure-time PA: objective	943	0.05	0.05	.05	−.10***	.18***	.01						
Active Travel: objective	1007	0.22	0.17	−.04	−.12***	−.08	−.00	.11					
BREQ-2 autonomous	1433	3.52	0.87	.47***	−.10**	.53***	.04	.05	.00				
BREQ-2 controlled	1433	2.34	0.85	.07	.35***	.10	−.02	−.06	−.04	.31***			
MATSS autonomous	1283	3.33	1.03	.43***	.02	.21***	−.04	.02	−.03	.53***	.27***		
MATSS controlled	1283	2.15	0.93	.01	.28***	−.08	−.07	.05	.03	.03	.65***	.23***	
BMI z-score	1177	7.00	1.09	−.04	.02	.02	.03	.05	−.01	.05	.13***	.05	.05

*BMI* Body Mass Index, *BREQ* Behavioral Regulation in Exercise Questionnaire, *MATSS* Motivation towards Active Travel to School Scale, *PA* physical activity. Affect and motivation were both measured on a Likert scale from 1 to 5

*p < .05, **p < .01, ***p < .001

To answer Research Question 4, we conducted latent variable moderation analyses to evaluate whether motivational constructs (i.e., autonomous motivation and controlled motivation) moderated the relationships between domain-specific PA and affective wellbeing. As shown in Additional file 3: Appendix C, we regressed the PA variable, the motivation variables, and the interaction terms onto the outcome variables (i.e., positive affect and negative affect). Again, we constrained corresponding interaction paths to be equal and conducted a Wald test to determine if the paths were significantly different from each other. We first conducted this procedure for leisure-time PA (Table 2) then for active travel (Table 3). Finally, we combined both PA domains in the same model (please see Additional file 4: Appendix D).

**Table 2:** Leisure-time Physical Activity and Affect: Structural Equation Model Testing Autonomous and Controlled Motivation as Moderators

	Unadjusted model				Adjusted model			
	β	*SE*	*p*	*R* ^2^	β	*SE*	*p*	*R* ^2^
Self-report model								
*Positive Affect*								
Leisure-time PA	−.02	.07	.76		−.09	.09	.30	
Autonomous motivation towards leisure-time PA	.51	.05	<.001		.55	.07	<.001	
Controlled motivation towards leisure-time PA	−.07	.04	.06		−.07	.03	.03	
Leisure-time PA × Autonomous Motivation	.05	.06	.45		.09	.07	.20	
Leisure-time PA × Controlled Motivation	.09	.03	.01		.03	.02	.07	
Age					.02	.01	.07	
Sex					.02	.01	.07	
SES					.02	.01	.07	
BMI					.04	.02	.07	
				.25***				.26***
*Negative Affect*								
Leisure-time PA	−.14	.06	.02		−.10	.05	.07	
Autonomous motivation towards leisure-time PA	−.12	.05	.019		−.11	.05	.03	
Controlled motivation towards leisure-time PA	.38	.05	<.001		.35	.06	<.001	
Leisure-time PA × Autonomous Motivation	.04	.04	.24		.03	.04	.41	
Leisure-time PA × Controlled Motivation	−.14	.06	.01		−.03	.01	.02	
Age					−.03	.01	.02	
Sex					−.02	.01	.02	
SES					−.03	.01	.02	
BMI					−.05	.02	.02	
				.19***				.15***
Objective model								
*Positive Affect*								
Leisure-time PA	.02	.03	.43		−.02	.04	.71	
Autonomous motivation towards leisure-time PA	.53	.04	<.001		.52	.03	<.001	
Controlled motivation towards leisure-time PA	−.06	.08	.47		−.07	.04	.12	
Leisure-time PA × Autonomous Motivation	−.02	.03	.62		−.01	.01	.29	
Leisure-time PA × Controlled Motivation	.02	.07	.83		.00	.00	.10	
Age					.03	.02	.10	
Sex					.03	.02	.10	
SES					.04	.03	.10	
BMI					.06	.04	.10	
				.30***				.26***
*Negative Affect*								
Leisure-time PA	−.07	.03	.03		−.04	.03	.13	
Autonomous motivation towards leisure-time PA	−.14	.04	<.001		−.12	.05	.01	
Controlled motivation towards leisure-time PA	.30	.09	<.01		.36	.06	<.001	
Leisure-time PA × Autonomous Motivation	−.02	.03	.64		−.01	.02	.57	
Leisure-time PA × Controlled Motivation	.06	.06	.36		−.00	.00	<.001	
Age					−.05	.01	<.001	
Sex					−.04	.01	<.001	
SES					−.05	.02	<.001	
BMI					−.10	.03	<.001	
				.14***				.15***

*BMI* Body Mass Index, *PA* physical activity, *SES* socioeconomic status. Adjusted model includes age, sex, socioeconomic status, and body mass index as covariates

*p < .05, **p < .01, ***p < .001

**Table 3:** Active Travel and Affect: Structural Equation Model Testing Autonomous and Controlled Motivation as Moderators

	Unadjusted model				Adjusted model			
	β	*SE*	*p*	*R* ^2^	β	*SE*	*p*	*R* ^2^
Self-report model								
*Positive Affect*								
Active travel	−.04	.04	.30		−.04	.03	.17	
Autonomous motivation towards active travel	.41	.06	<.001		.45	.05	<.001	
Controlled motivation towards active travel	.00	.07	.98		−.11	.06	.06	
Active Travel × Autonomous Motivation	.02	.03	.47		−.01	.03	.70	
Active Travel × Controlled Motivation	−.08	.05	.11		.01	.01	.09	
Age					.02	.02	.12	
Sex					.02	.01	.12	
SES					.03	.02	.12	
BMI					.05	.03	.12	
				.19***				.19***
*Negative Affect*								
Active travel	.01	.03	.73		.02	.03	.53	
Autonomous motivation towards active travel	−.06	.06	.37		−.10	.09	.26	
Controlled motivation towards active travel	.19	.06	<.01		.29	.04	<.001	
Active Travel × Autonomous Motivation	−.01	.03	.87		.04	.04	.30	
Active Travel × Controlled Motivation	.08	.04	.03		−.01	.01	.19	
Age					−.02	.01	.22	
Sex					−.02	.01	.22	
SES					−.02	.01	.22	
BMI					−.03	.01	.22	
				.09**				085***
Objective model								
*Positive Affect*								
Active travel	−.02	.03	.39		−.01	.03	.65	
Autonomous motivation towards active travel	.28	.06	<.001		.28	.08	<.001	
Controlled motivation towards active travel	−.11	.11	.36		−.08	.07	.23	
Active Travel × Autonomous Motivation	.09	.03	<.01		.09	.04	.03	
Active Travel × Controlled Motivation	.04	.05	.42		.01	.00	.05	
Age					.03	.02	.07	
Sex					.03	.02	.07	
SES					.04	.02	.07	
BMI					.06	.03	.07	
				.17***				.17***
*Negative Affect*								
Active travel	−.12	.03	<.001		−.11	.03	<.001	
Autonomous motivation towards active travel	.02	.06	.84		.03	.09	.71	
Controlled motivation towards active travel	.34	.12	<.01		.27	.06	<.001	
Active Travel × Autonomous Motivation	−.02	.03	.37		−.03	.03	.35	
Active Travel × Controlled Motivation	−.05	.05	.37		−.01.	.00	<.001	
Age					−.04	.01	<.001	
Sex					−.04	.01	<.001	
SES					−.04	.02	<.001	
BMI					.06	.03	.07	
				.09*				.09**

*BMI* Body Mass Index, *PA* physical activity, *SES* socioeconomic status. Adjusted model includes age, sex, socioeconomic status, and body mass index as covariates

*p < .05, **p < .01, ***p < .001

## Results

### Descriptive statistics

Participants (55% male and 45% female) were 11 to 15 years old (*M* = 12.94, *SD* = 0.54) and were mostly born in Australia (72.75%), with 8.67% identifying their background as ‘Indigenous Australian.’ Half of the participants were within a healthy weight range (50.69%), with 17.36% overweight, and 7.63% obese. On average, adolescents self-reported being moderately or vigorously active during their leisure-time three-days per week. Accelerometer data indicated that on average students spent 66.9 min per day in MVPA during leisure-time (*SD* = 55.25). Adolescents self-reported that half of their trips to or from school included active travel (*M* = 50%) and according to accelerometer data, 22% of the students’ recalled travel time was spent in objectively-measured MVPA. Students who engaged in active travel to school accumulated 15.8 min of activity per trip according to the self-report data, and 7.8 min of MVPA per trip according to accelerometer data.

Participants’ scores on the positive (*M* = 3.64, *SD* = 0.89) and negative affect scales (*M* = 1.89, *SD* = 0.84) were similar to previously reported outcomes for this age-group [42], as were scores on the motivation scales [43]. Autonomous motivation towards leisure-time PA (*r* = .47) and active travel (*r* = .43) were positively correlated with positive affect, while controlled motivation towards leisure-time PA (*r* = .35) and active travel (*r* = .28) were positively correlated with negative affect.

### Main effects

#### Leisure-time physical activity

Results of the initial model including only PA and wellbeing variables indicated that self-reported leisure-time PA had a moderate positive association with positive affect (β = .29, *p* < .001) and a small-to-moderate statistically significant inverse association with negative affect (β = −.19, *p* < .001). Accelerometer-measured leisure-time PA was not significantly associated with positive affect (β = .05, *p* > .05) but, was inversely associated with negative affect (β = −.09, *p* < .05).

#### Active travel

The initial model including only PA and wellbeing variables indicated that self-reported active travel was not associated with positive affect (β = −.06, *p* > .05), and this association was significantly weaker (*p* < .001) than the relationship between self-reported leisure-time and positive affect. Self-reported active travel was also not associated with negative affect (β = .00, *p* > .05). Accelerometer-measured active travel had no association with positive affect (β = −.06, *p* > .05) and a weak statistically significant negative association with negative affect (β = −.11, *p* < .001).

#### Motivation and affect

Autonomous motivation towards leisure-time PA was positively associated with positive affect in the self-report (β = .51, *p* < .001) and objective (β = .53, *p* < .001) model, and inversely associated with negative affect in the self-report (β = −.12, *p* < .05) and objective (β = −.14, *p* < .001) model (Table 2). Controlled motivation towards leisure-time PA was positively associated with negative affect in the self-reported (β = .38, *p* < .001) and objective (β = .30, *p* < .01) model. Autonomous motivation towards active travel was positively associated with positive affect in the self-report (β = .41, *p* < .001) and objective (β = .28, *p* < .001) model (Table 3). Controlled motivation towards active travel was positively associated with negative affect in both the self-report (β = .19, *p* < .01) and objective (β = .34, *p* < .01) model.

### Moderation effects

#### Leisure-time physical activity

As shown in the adjusted results in Table 2, neither autonomous nor controlled motivation significantly moderated the relationship between leisure-time PA and positive affect, in either the self-report or objective data.

In terms of negative affect, controlled motivation significantly moderated (β = −.03, *p* < .05) the relationship between self-reported leisure-time PA and negative affect in the adjusted model. However, the effect appeared negligible. Evaluating the interaction at one *SD* above and below the mean for controlled motivation revealed that the relationship between leisure-time PA and negative affect at one *SD* above the mean was β = −.13, and at one *SD* below the mean was β = −.07. Controlled motivation also significantly moderated the relationship between leisure-time PA and negative affect (β = −.003, *p* < .001) in the objective adjusted model. However, again, the interaction effect was small, and the relationship did not vary between one *SD* above (β = −.04) and below (β = −.04) the mean.

#### Active travel

As shown in Table 3, in the adjusted model, we did not find any significant moderator effects in the self-report data. However, in the objective model, autonomous motivation was a significant positive moderator (β = .09, *p* < .05) of the relationship between active travel and positive affect, meaning that the relationship between active travel and positive affect was more positive when autonomous motivation was higher. More specifically, when evaluating the interaction at one *SD* above the mean on autonomous motivation, a positive relationship was present between accelerometer-measured active travel and positive affect (β = .09, *p* < .01). Evaluating the interaction at one *SD* below the mean revealed an inverse relationship between accelerometer-measured active travel and positive affect (β = −.07, *p* < .01). As such, the direction of the relationship between accelerometer-assessed active travel and positive affect appears to be contingent upon the level of autonomous motivation.

In terms of negative affect, in the objective adjusted model, controlled motivation significantly moderated the relationship between active travel and negative affect (β = −.01, *p* < .001). However, this effect appeared negligible as evaluating the interaction one *SD* above and below the mean revealed very little variation in the relationship between active travel and negative affect for those one *SD* above the mean (β = −.12), compared to those one *SD* below the mean (β = −.10).

## Discussion

Walking is universally regarded as the ideal form of PA [44], and given the challenges of increasing participation in leisure-time PA [16], many recent PA guidelines and campaigns encourage incidental lifestyle PA, such as walking to school or work [45–47]. This approach may increase PA and improve physical health in some cases [48]; however, PA outside leisure-time is not as likely to benefit mental health [7] and there is increasing evidence that guidelines need to distinguish between different PA domains [48]. If PA is to be a truly valuable approach to promoting mental health among young people, then either leisure-time PA may need to be prioritized above other PA domains, or research needs to determine under which circumstances PA outside leisure-time is, and is not, positively associated with mental health. This study examined whether motivation influences the strength of the relationship between PA and mental wellbeing within leisure-time and active travel.

Our results showed that autonomous motivation towards leisure-time PA had a strong relationship with self-reported leisure-time PA (*r* = .53), consistent with the strength of association reported in previous studies also using self-report measures [49]. However, there was no relationship between autonomous motivation towards leisure-time PA and accelerometer-assessed leisure-time PA (*r* = .05). This finding is consistent with previous work, as studies examining the relationship between autonomous motivation and accelerometer assessed leisure-time PA typically report weaker relationships, compared to when leisure time PA is self-reported [50]. Neither autonomous motivation towards active travel, nor controlled motivation towards active travel, were associated with self-reported or accelerometer-assessed active travel. Hence, the relationships between motivation and PA were inconsistent between PA domains and between different measures of PA. The relationship between motivation towards active travel and active travel behavior had not yet been investigated but, the lack of a relationship suggests that motivation towards active travel may not influence the amount of active travel adolescents undertake. As such, motivation appears to play different roles in different PA domains.

Autonomous motivation was consistently positively associated with positive affect, and inversely related to negative affect, across both PA domains – meaning that, although autonomous motivation towards active travel was not associated with active travel behavior, it was associated with affective wellbeing. While the size of these relationships may be inflated due to common method variance [51]; previous studies in physical education, leisure-time, and elite sport contexts, have also shown that autonomous motivation is associated with positive affective outcomes [18–20, 52]. Thus, autonomous motivation itself may independently contribute to people’s affective wellbeing. For adolescents to internalize motivation towards a behavior, they must have the perception that their social contexts and environments enable them to function or behave autonomously [53]. Therefore, those who report autonomous motivation towards PA may naturally have better psychological wellbeing due to the perception that they function autonomously, regardless of how often they actually participate in the behavior [53].

Examining the relationships between domain-specific PA and wellbeing only, to answer Research Questions 1–3, showed that leisure-time PA was a stronger predictor of affective wellbeing than active travel. This finding is consistent with previous studies that have shown, in comparison to active travel, leisure-time PA is more strongly associated with reduced depression [27] and negative affect [14], and increased mental wellbeing [54]. Although there are a number of physiological explanations as to why exercise is associated with improved mood, these hypotheses are often criticized for oversimplifying the effect of exercise [55]. Psychological hypotheses however, explain that distracting individuals from daily stressors, providing positive social interactions, and increasing one’s self-esteem may contribute to the wellbeing benefits often associated with PA [55, 56]. Perhaps, active travel does not provide a distraction from stress or opportunities for social interaction and improved self-esteem to the same degree as typical leisure-time activities (e.g., team sport), and, thereby, leisure-time is more likely to be associated with wellbeing.

By adding motivation to the model as a moderator we answered Research Question 4, and in doing so, identified that while motivation is associated with increased leisure-time PA, it does not appear to moderate the effect of the leisure-time PA on wellbeing. In contrast, for active travel, motivation may not be associated with participation, but instead, may influence outcomes relating to mental health. It may be the case that leisure-time innately provides adolescents with perceived choice over the way in which they spend their time. Therefore, those who are autonomously motivated towards leisure-time PA are more physically active (see Table 1). However, the majority of students have little control over whether they actively or passively travel to and from school [25]; causing motivation to not influence behavior but to potentially impact wellbeing.

According to self-determination theory, the reason that motivation influences mental wellbeing is because activities which individuals are usually autonomously motivated towards are usually activities which satisfy their basic psychological needs for autonomy (the desire to experience volition by being the origin of, and self-endorsing, one’s behavior), competence (experiencing a sense of confidence and feeling effective while expressing one’s capabilities), and relatedness (the fundamental need to maintain close personal connections and feel like a valuable and cared for member of a group) [57–60]. Therefore, students who are autonomously motivated towards active travel could potentially receive psychological benefits from experiencing a sense of autonomy when travelling without parental supervision, a sense of competence from the activity they have undertaken, or feeling connected to peers or siblings with whom they actively travel. It may be the case that the majority of leisure-time PA experiences (e.g., organized sport) satisfy adolescents’ psychological needs, whereas, for some adolescents, active travel supports their needs, and for others their needs are thwarted by feeling forced.

In domains where, compared with leisure-time, the choice to participate in PA is often constrained (e.g., active travel, work, household duties) it may be particularly important to ensure that activities fulfill basic psychological needs in other ways. For example, ensuring that PA optimally challenges individuals (i.e., satisfying the need for competence), fosters an interpersonally supportive environment (i.e., satisfying the need for relatedness), and highlights the health benefits of PA (i.e., providing a rationale to fulfill the need for autonomy). Adopting such approaches could shift people’s locus of control to a more internal focus, resulting in more autonomous motivation and increased affective wellbeing, despite the individual originally participating for controlled reasons [16].

Despite the novelty of findings produced through this research, the effect sizes for active travel were small, and the same results were not identified for leisure-time. These results are certainly interesting, and could lead to better promotion of mental health through PA, but are preliminary at this stage. Findings need to be replicated in similar samples, and research needs to investigate the role of motivation in a broader number of PA domains. Further, motivation and PA need to be further examined together to truly determine the role each variable plays in enhancing wellbeing. It is also important to recognize that mental health could influence people’s participation in PA. Indeed, Teychenne et al. [61] demonstrated that depressive symptoms predicted low levels of leisure-time PA in the future, while active travel predicted future risk of depression. As such, the direction of the relationship between PA and mental health may also vary between PA domains and therefore, the examination of motivation, PA, and mental health needs to be explored through longitudinal and experimental designs. Also, given that motivation did not play the same role in the relationship between leisure-time PA and affect, as it did for active travel, further investigation of this domain is warranted. Examining whether the amount of PA mediates the direct relationship between motivation and affect could provide valuable insights in terms of understanding leisure-time PA. Lastly, motivation, wellbeing, and PA could be measured on a daily basis to enhance understanding as moderation may play a larger role at the within-person level (e.g., between different activities, on different days, when motivation is more or less autonomous) than between individuals.

### Strengths and limitations

This study is the first to assess whether motivation moderates the relationships between domain-specific PA and affective wellbeing. The large sample size and the use of accelerometers are strengths. Although using accelerometers is a strength, our accelerometer measure of active travel still included a self-report component (i.e., reporting departure and arrival times). Using global positioning systems or wearable cameras together with accelerometers may enable researchers to obtain more accurate assessments of domain-specific PA [4, 62]. While our sample included both genders and a variety of cultures, results may not be generalizable to older adolescents or children, or to those living in higher socio-economic areas. We also had a higher than usual proportion of students classified as underweight. Because we collected data cross-sectionally, we cannot infer that increased participation in autonomously motivated physical activity led to increased affective wellbeing. Finally, using different measures of motivation in different contexts limits the degree which one can compare contexts. As it is impossible to know how our results are influenced by the use of two different measures of motivation, we have made no attempts to draw specific comparisons between PA domains in terms of the participants’ levels of motivation. Nevertheless, future research should seek to identify identical items across contexts. It would also be a valid approach for future research to use the same measure of motivation across contexts, and compare results to our current approach using different measures.

## Conclusions

Current national and international guidelines focus on the duration, type, and intensity of PA that adolescents need to undertake. Our findings suggest that the life domain in which PA occurs, and potentially the reasons why they participate, may have implications for wellbeing. Given that poor mental health is the leading contributor to disease burden among adolescents [63], PA guidelines should consider acknowledging the importance of leisure-time PA, ahead of PA in other life domains. However, given the difficulty of increasing participation in leisure-time PA [16], understanding how to improve the effect of PA undertaken during other domains is also imperative. Although active travel is not as strongly associated with affective wellbeing as leisure-time PA, adolescents who autonomously walk or cycle to school appear more likely to experience positive affect while those who feel forced or pressured may experience negative affect. As such, finding ways to enhance autonomous motivation towards active travel is also an important endeavor.

## Additional files

Additional file 1: **Appendix A**. Travel Diary. (PDF 210 kb)

Additional file 2: **Appendix B**. The Motivation towards Active Travel to School Scale (MATSS): Instrument development and initial validity evidence. (DOCX 61 kb)

Additional file 3: **Figure C1***.* Self-reported leisure-time physical activity and affect: Structural equation model testing autonomous and controlled motivation as moderators. **Figure C2***.* Objectively measured leisure-time physical activity and affect: Structural equation model testing autonomous and controlled motivation as moderators. **Figure C3***.* Self-reported active travel and affect: Structural equation model testing autonomous and controlled motivation as moderators. **Figure C4***.* Objectively measured active travel and affect: Structural equation model testing autonomous and controlled motivation as moderators. (DOCX 310 kb)

Additional file 4: **Table D1.** Domain-Specific Physical Activity and Affect: Structural Equation Model Testing Autonomous and Controlled Motivation as a Moderators. (DOCX 21 kb)

## Abbreviations

MVPA: Moderate to vigorous physical activity
PA: Physical activity
