# Supplementary material for: Domain-specific physical activity and affective wellbeing among adolescents: an observational study of the moderating roles of autonomous and controlled motivation
Source: Int J Behav Nutr Phys Act. 2018 Sep 10;15:87. doi: 10.1186/s12966-018-0722-0 (PMC6131748; doi:10.1186/s12966-018-0722-0)
Supplement: Supplementary file 1 — Appendix A. Travel Diary. (PDF 210 kb) [file 12966_2018_722_MOESM1_ESM.pdf]

Appendix A: Travel Diary

MONDAY

1. Please answer <u>as accurately as you can</u> about your trip <u>TO SCHOOL</u> on Monday morning.

What time did you leave home? Hour ✐………… Minute✐…………

What time did you arrive at school? Hour ✐………… Minute✐…………

Please fill in the following table based on how you got <u>TO</u> school and how long it took.

	You may colour MORE THAN ONE (●)	How many minutes did you spend in each type of transport?
**Walk**	○	✐…………hours and ✐…………minutes
**Bicycle**	○	✐…………hours and ✐…………minutes
**Skateboard/Scooter**	○	✐…………hours and ✐…………minutes
**Bus**	○	✐…………hours and ✐…………minutes
**Train**	○	✐…………hours and ✐…………minutes
**Car**	○	✐…………hours and ✐…………minutes
**Other**	✐………………………	✐…………hours and ✐…………minutes

2. Please answer <u>as accurately as you can</u> about your trip <u>FROM SCHOOL</u> on Monday afternoon.

What time did you leave school? Hour ✐………… Minute✐…………

What time did you arrive at your next destination? Hour ✐………… Minute✐…………

Was this destination home Ⓗ or other Ⓞ

If other, please explain

………..…………………………………………………………………………… ✐

Please fill in the following table based on your trip FROM school and how long it took.

	You may colour MORE THAN ONE (●)	How many minutes did you spend in each type of transport?
**Walk**	○	✐………..hours and ✐………..minutes
**Bicycle**	○	✐………..hours and ✐………..minutes
**Skateboard/Scooter**	○	✐………..hours and ✐………..minutes
**Bus**	○	✐………..hours and ✐………..minutes
**Train**	○	✐………..hours and ✐………..minutes
**Car**	○	✐………..hours and ✐………..minutes
**Other**	✐………………………	✐………..hours and ✐………..minutes
