# Supplementary material for: Domain-specific physical activity and affective wellbeing among adolescents: an observational study of the moderating roles of autonomous and controlled motivation
Source: Int J Behav Nutr Phys Act. 2018 Sep 10;15:87. doi: 10.1186/s12966-018-0722-0 (PMC6131748; doi:10.1186/s12966-018-0722-0)
Supplement: Supplementary file 2 — Appendix B. The Motivation towards Active Travel to School Scale (MATSS): Instrument development and initial validity evidence. (DOCX 61 kb) [file 12966_2018_722_MOESM2_ESM.docx]

**Appendix B: The Motivation towards Active Travel to School Scale (MATSS): Instrument development and initial validity evidence**

This Appendix details the methods and results of a preliminary study that we undertook to develop and test the psychometric properties of the Motivation towards Active Travel to School Scale (MATSS). We developed the MATSS to measure motivation towards adolescents’ active travel to and from school, in order to test whether motivation moderated the relationships between active travel and affective wellbeing.

**Introduction**

Self-determination theory has been used as a framework to examine motivation towards a variety of behaviours, including: school and tertiary education, attendance at health-related appointments (e.g., dentist), smoking cessation, injury rehabilitation, weight-loss maintenance, and medication adherence; television watching, internet gaming, and social media, and; exercise, sport, and physical education. As such, researchers have developed abundant questionnaires to measure motivation within specific contexts. For example, the Academic Motivation Scale [[1](#_ENREF_1)], the Science Motivation Questionnaire-II [[2](#_ENREF_2)], the Strength of Motivation for Medical School scale [[3](#_ENREF_3)], and the Motivation Scale of English Learning [[4](#_ENREF_4)] all measure motivation within different education settings. Similarly, the Exercise Motivations Inventory measures exercise motivation [[5](#_ENREF_5)], while the Behavioral Regulation in Sport Questionnaire measures sport motivation [[6](#_ENREF_6)], the BREQ-2 measures leisure-time PA motivation [[7](#_ENREF_7)], and the Perceived Locus of Causality Questionnaire measures physical education motivation [[8](#_ENREF_8)]. Yet, no measure exists specifically for active travel.

Although the BREQ-2 is one of the most widely used measures of exercise motivation, and has good psychometric properties when used to assess motivation towards leisure-time PA [[9](#_ENREF_9)], motivation is likely to manifest differently in leisure-time compared to active travel. Qualitative evidence shows that adolescents participate in leisure-time PA and active travel for different reasons [[10](#_ENREF_10)]. Given the way in which motivation manifests itself is context-specific, it is standard across the field for studies to use different measures of motivation in different contexts. Using different measures of motivation in different contexts does limit the degree which one can compare contexts. However, using the same questionnaire to measure motivation in two different contexts where motivation may not manifest in the same way is also limiting. For example, Chan, Lonsdale, and Fung [[11](#_ENREF_11)] examined the influence of coaches, parents, and peers on athlete’s motivation using the same measure for each social agent but, recognised this method as a limitation as using identical items across contexts does not account for the unique aspects of each social agent. Indeed, Keegan, Harwood, Spray, and Lavallee [[12](#_ENREF_12)] identified that different social agents play different roles in manifesting motivation. Just as motivational influences are manifested differently by peers and parents, motivation may manifest differently in leisure-time and active travel. Therefore, it is not appropriate to measure motivation in different contexts the same way and the purpose of this study was to develop a measure of motivation specifically towards active travel to school among adolescents.

**Phase 1: Item Development**

**Methods**

The first step in developing the Motivation towards Active Travel to School Scale (MATSS) involved creating initial items based on the conceptual definitions of autonomous motivation, controlled motivation, and amotivation, as outlined in the introduction of the main text. In order to generate items specifically related to active travel, we examined qualitative evidence of adolescents’ reported motivation towards active travel [[10](#_ENREF_10)]. We also looked to the BREQ-2 [[7](#_ENREF_7)], PLOC [[8](#_ENREF_8)], and MLSQ [[13](#_ENREF_13)], as well as conceptual definitions from self-determination theory [[14](#_ENREF_14)] to ensure that items covered key elements of each construct. We intended that the instrument would be brief, between nine and 12 items in length, so that the measure could be administered alongside other scales [[15](#_ENREF_15)]. We created an initial item pool of 28 items, as we expected some items would be not be retained during the development process [[16](#_ENREF_16)]. Ten items were designed to measure autonomous motivation (items 1-5 related to intrinsic motivation and items 6-10 related to identified regulation), 11 controlled motivation (items 11-15 related to introjected regulation and items 16-21 related to external regulation), and seven amotivation (items 22-28). Next, 25 researchers who had published peer reviewed articles in the area of self-determination theory (i.e., expert judges), rated the degree of match between each of the 28 items and the three motivation constructs on a 5-point Likert scale, where 1 indicated a *poor match* and 5 represented an *excellent match* [[17](#_ENREF_17)].

***Data Analysis.*** We calculated Mahalanobis distances to identify expert raters whose scores represented multivariate outliers. We also calculated the mean, standard deviation, and Aiken’s item content-validity coefficient (*V*) for each item (see Table B1). *V* indicated the relevance of each item to each construct and, with 25 expert judges, *V* values are significant at *p* < .05 for *V* = .63 [[17](#_ENREF_17), [18](#_ENREF_18)]. We then calculated Cohen’s effect size indices (see Table B2) to indicate the magnitude of difference between the mean expert rating for each item on each of the three subscales [[19](#_ENREF_19)]. A large effect size (≥ .80) indicated a large difference between the mean rating for that item on two different subscales (i.e., the subscale it was intended to measure and a subscale to which it should not be theoretically related). Therefore, effect sizes ≥ .80 indicate that the item was only relevant to the intended subscale.

**Results**

All experts’ Mahalanobis distances were lower than the critical value of 20.52; thus, no experts’ ratings were removed from analyses [[20](#_ENREF_20)]. The validity coefficients of 24 of the 28 initial items were significant (*V ≥* .63, *p* < .05) and were retained. Cohen’s *d* values for all 24 retained items were ≥ .80, suggesting that each item was only relevant to the construct it was intended to measure. Given that the number of items with acceptable content validity (i.e., 24) was larger than the number of items needed for the final MATSS, we removed another 8 items. The items we removed had either a lower *V* coefficient compared to the retained items in the same subscale, or negative comments provided by the self-determination theory experts with regards to the wording of the item. Some of the initial items developed that were similar to BREQ-2 items were not suitable for assessing motivation towards active travel. For example, the item “because I feel under pressure from other people (e.g., family, friends)” is very similar to the BREQ-2 item “I feel under pressure from my friends/family to exercise” but, had one of the lowest *V* coefficients out of all potential items for the controlled subscale, and a smaller effect size, compared to other potential items (reflecting less agreement between the experts in terms of how the item related to the intended construct). Therefore, this item was not retained for analyses in Phase II. The 16 retained items collectively covered all components of the three constructs (i.e., autonomous motivation, controlled motivation, and amotivation).

Table B1

*Mean ratings and content validity coefficients of the initial items.*

|  | **Intended Construct** | **Autonomous Motivation** | | | **Controlled Motivation** | | | **Amotivation** | | |
| --- | --- | --- | --- | --- | --- | --- | --- | --- | --- | --- |
| **Item** |  | ***M*** | ***SD*** | ***V*** | ***M*** | ***SD*** | ***V*** | ***M*** | ***SD*** | ***V*** |
| 1. **Because it is fun.** | AUT | 4.92 | .28 | **.93** | 1.00 | .00 | .00 | 1.00 | .00 | .00 |
| 1. **Because I enjoy it.** | AUT | 4.92 | .28 | **.93** | 1.00 | .00 | .00 | 1.00 | .00 | .00 |
| 1. Because I’ll miss out on an enjoyable activity if I don’t. | AUT | 3.00 | 1.26 | .50 | 2.12 | 1.24 | .28 | 1.24 | .72 | .06 |
| 1. **Because I find it pleasurable.** | AUT | 4.52 | 1.05 | **.88** | 1.08 | .28 | .02 | 1.00 | .00 | .00 |
| 1. **Because it is interesting.** | AUT | 4.76 | .52 | **.94** | 1.00 | .00 | .00 | 1.00 | .00 | .00 |
| 1. **Because it is personally important to me.** | AUT | 4.71 | .62 | **.88** | 1.28 | .54 | .07 | 1.00 | .00 | .00 |
| 1. **Because I think it’s a worthwhile thing to do.** | AUT | 4.12 | 1.05 | **.78** | 1.46 | .78 | .10 | 1.00 | .00 | .00 |
| 1. Because if I don’t, I miss out on personally valuable benefits. | AUT | 3.40 | 1.26 | .60 | 2.08 | 1.15 | .27 | 1.08 | .40 | .02 |
| 1. **Because the benefits are important to me.** | AUT | 4.00 | 1.18 | **.71** | 1.60 | .96 | .15 | 1.00 | .00 | .00 |
| 1. **Because I think it is good for me.** | AUT | 3.56 | 1.08 | **.64** | 1.92 | 1.12 | .23 | 1.00 | .00 | .00 |
| 1. **Because I feel guilty if I don’t.** | CON | 1.16 | .62 | .04 | 4.92 | .28 | **.98** | 1.00 | .00 | .00 |
| 1. **Because I feel bad about myself if I don’t.** | CON | 1.00 | .00 | .00 | 4.88 | .33 | **.97** | 1.04 | .20 | .01 |
| 1. **Because I feel lazy if I don’t.** | CON | 1.08 | .28 | .02 | 4.08 | 1.35 | **.77** | 1.16 | .47 | .04 |
| 1. **Because I need to do it to feel good about myself.** | CON | 1.32 | .63 | .08 | 4.24 | .78 | **.81** | 1.04 | .20 | .01 |
| 1. **Because it makes me feel worthy as a person.** | CON | 1.44 | .96 | .11 | 3.92 | 1.08 | **.73** | 1.04 | .20 | .01 |
| 1. **Because other people (e.g., parents, friends) get angry with me if I don’t.** | CON | 1.00 | .00 | .00 | 4.80 | .50 | **.95** | 1.08 | .40 | .02 |
| 1. **Because other people (e.g., parents, friends) get upset with me if I don’t.** | CON | 1.00 | .00 | .00 | 4.60 | .91 | **.90** | 1.08 | .40 | .02 |
| 1. **Because other people (e.g., parents, friends) tell me I should.** | CON | 1.00 | .00 | .00 | 4.56 | .92 | **.89** | 1.04 | .20 | .01 |
| 1. **Because other people (e.g., parents, friends) force me to.** | CON | 1.00 | .00 | .00 | 4.56 | 1.04 | **.89** | 1.12 | .60 | .03 |
| 1. **Because I feel under pressure from other people (e.g., parents, friends).** | CON | 1.00 | .00 | .00 | 4.64 | .95 | **.91** | 1.08 | .40 | .02 |
| 1. **Because other people (e.g., parents, friends) pressure me.** | CON | 1.00 | .00 | .00 | 4.52 | 1.16 | **.88** | 1.12 | .60 | .03 |
| 1. Because there is no other way for me to get to school. | AMO | 1.12 | .33 | .03 | 2.44 | 1.29 | .36 | 2.00 | 1.19 | .25 |
| 1. Because I have no choice. | AMO | 1.12 | .60 | .03 | 2.84 | 1.28 | .46 | 2.12 | 1.27 | .28 |
| 1. **And there may be good reason to, but I personally don’t see any.** | AMO | 1.04 | .20 | .01 | 1.12 | .33 | .03 | 4.04 | 1.34 | **.76** |
| 1. **But I don’t see why I should.** | AMO | 1.04 | .20 | .01 | 1.16 | .47 | .04 | 4.36 | 1.15 | **.84** |
| 1. **But I feel it is a waste of time.** | AMO | 1.00 | .00 | .00 | 1.08 | .28 | .02 | 4.20 | 1.35 | **.80** |
| 1. **But I don’t see the point.** | AMO | 1.08 | .40 | .02 | 1.08 | .28 | .02 | 4.28 | 1.21 | **.82** |
| 1. **But I’m not sure if it’s worth it.** | AMO | 1.00 | .00 | .00 | 1.20 | .50 | .05 | 4.08 | 1.29 | **.77** |

*Note.* Retained items are bolded. *V* = Aiken’s item content-validity coefficient; AUT = Autonomous motivation; CON = Controlled Motivation, and AMO = Amotivation.

Table B2

*Effect size indices of the initial items.*

| **Item** | **Autonomous**  **vs**  **Controlled** | **Controlled**  **vs**  **Amotivation** | **Amotivation**  **vs**  **Autonomous** |
| --- | --- | --- | --- |
| 1. **Because it is fun.** | **18.48** | **-** | **18.48** |
| 1. **Because I enjoy it.** | **18.48** | **-** | **18.48** |
| 1. **Because I find it pleasurable.** | **5.20** | **0.38** | **4.98** |
| 1. **Because it is interesting.** | **10.63** | **-** | **10.63** |
| 1. **Because it is personally important to me.** | **6.21** | **0.79** | **8.74** |
| 1. **Because I think it’s a worthwhile thing to do.** | **2.77** | **0.81** | **4.01** |
| 1. **Because the benefits are important to me.** | **2.17** | **0.85** | **3.54** |
| 1. **Because I think it is good for me.** | **1.49** | **1.18** | **3.29** |
| 1. **Because I feel guilty if I don’t.** | **8.03** | **19.80** | **0.38** |
| 1. **Because I feel bad about myself if I don’t.** | **16.63** | **14.07** | **0.28** |
| 1. **Because I feel lazy if I don’t.** | **3.07** | **2.89** | **0.20** |
| 1. **Because I need to do it to feel good about myself.** | **4.23** | **5.62** | **0.58** |
| 1. **Because it makes me feel worthy as a person.** | **2.38** | **3.71** | **0.55** |
| 1. **Because other people (e.g., parents, friends) get angry with me if I don’t.** | **10.75** | **8.22** | **0.28** |
| 1. **Because other people (e.g., parents, friends) get upset with me if I don’t.** | **5.59** | **5.01** | **0.28** |
| 1. **Because other people (e.g., parents, friends) tell me I should.** | **5.47** | **5.29** | **0.28** |
| 1. **Because other people (e.g., parents, friends) force me to.** | **4.84** | **4.05** | **0.28** |
| 1. **Because I feel under pressure from other people (e.g., parents, friends).** | **5.42** | **1.22** | **0.03** |
| 1. **Because other people (e.g., parents, friends) pressure me.** | **4.29** | **3.68** | **0.28** |
| 1. **And there may be good reason to, but I personally don’t see any.** | **0.29** | **2.99** | **3.13** |
| 1. **But I don’t see why I should.** | **0.33** | **3.64** | **4.02** |
| 1. **But I feel it is a waste of time.** | **0.40** | **3.20** | **3.35** |
| 1. **But I don’t see the point.** | **0.00** | **3.64** | **3.55** |
| 1. **But I’m not sure if it’s worth it.** | **0.57** | **2.94** | **3.38** |

*Note.* Effect sizes = Cohen’s *d* [[21](#_ENREF_21)]. Retained items are bolded. Where all experts’ rated the match between an item and a subscale the same (e.g., 1 poor match) and *SD* = 0, an effect size could not be calculated and is represented with a dash.

**Phase 2: Initial Validity Evidence**

**Methods**

In the second phase of developing the MATSS we used confirmatory factor analysis (CFA) to determine the relationship between scores derived from each item and the latent variable it represented [[16](#_ENREF_16)].

***Participants.*** A sample of 239 boys aged 12-15 years (*M* = 13.25, *SD* = 0.67) from two non-government high schools in Sydney, Australia completed the 16-item MATSS. Ethics approval was received and each student provided parental consent and participant assent.

***Measure.*** Students first read the following explanation of active travel; “active travel to or from school means that you travel at least part of your journey by: walking, cycling, scooter, skateboarding, or some other kind of physical activity. Active travel includes trips when only part of your journey was active. For example, you might walk to catch a bus” [[22](#_ENREF_22), [23](#_ENREF_23)]. The MATSS then consisted of the stem “I actively travel to or from school...” and was followed by 16 items (i.e., six items measuring autonomous motivation, five measuring controlled motivation, and five measuring amotivation). Participants then responded to each item on a 5-point Likert scale from *strongly disagree* = 1 to *strongly agree* = 5.

***Data Analysis.*** To account for the 1.54% of the MATSS data points missing, we employed full information maximum likelihood estimation. To account for potential violations of multivariate normality, we used robust standard errors (MLR). First, we investigated the factor structure of the full 16-item model using CFA in MPlus (version 7.4) [[24](#_ENREF_24)]. However, as a briefer version of the measure was desirable, we removed further items based on low item-factor loadings (<.40), large standardized residuals (>2), or high modification indices [[25](#_ENREF_25)]. We again utilised CFA to assess the construct validity of the revised model by testing whether the data fit the hypothesized three-factor model (autonomous motivation, controlled motivation, amotivation). A comparative fit index (CFI) close to .95, a standardised root mean square residual (SRMR) ≤.08, and a root mean squared error of approximation (RMSEA) close to .06 indicate very good fit [[26](#_ENREF_26)]. Interfactor correlations (i.e., correlations between the three latent motivation scores) were also calculated to assess the nomological network (i.e., when stronger correlations exist between factors that lie closer together on the self-determination theory continuum). Finally, we used Raykov’s formula, ρ = (Σλ)²/[(Σλ)²+(ΣΘ)], where λ = item factor loadings and Θ = error terms, to assess composite reliability (i.e., internal consistency) [[27](#_ENREF_27)]. Raykov’s ρ is similar to the commonly used Cronbach’s coefficient α, yet it uses a structural equation model to estimate composite reliability and does not possess the tendency to underestimate reliability that is found with Cronbach’s coefficient α [[27](#_ENREF_27), [28](#_ENREF_28)].

**Results**

Initial analysis of scores from the 16 items revealed poor fit of the data to the hypothesized three factor model (CFI = .87, SRMR = .09, RMSEA = .09). This poor fit was largely due to seven items cross loading onto two motivation subscales. These items also had the lowest factor loadings on their intended subscale. For example, the item “because it’s personally important to me” is similar to the BREQ-2 item “it’s important to me to exercise regularly” but, had a large modification index, suggesting the item cross loaded onto both the controlled motivation and amotivation subscales. As such, this item was not a good measure of autonomous motivation towards active travel despite being a good item for assessing autonomous motivation towards exercise. To reduce the length of the scale and increase the factorial validity of the MATSS scores, we removed the seven items with high modification indices. The retained items measured the intended constructs (as indicated by strong factor loadings) and had minimal cross loadings (as indicated by low modification indices). A subjective evaluation of the three items retained in each subscale indicated that the nine items maintained the breadth of content of the 16-item model [[25](#_ENREF_25)].

Analysis of the revised 9-item model showed good fit to the data (χ² = 47.71, CFI = .95, SRMR = .07, and RMSEA = .06; Table B3). The factor loadings ranged from .57 to .87 for the autonomous motivation subscale, .36 to .99 for the controlled motivation subscale, and .69 to .87 for the amotivation subscale (Table B4). Negative interfactor correlations were found between autonomous and controlled motivation (*r* = -.15, *p* = .03) and between autonomous motivation and amotivation (*r* = -.51, *p* < .001), and a positive correlation was found between controlled motivation and amotivation (*r* = .22, *p* < .01). The composite reliability scores were ρ = .77 for the autonomous motivation subscale, ρ = .61 for controlled motivation, and ρ = .83 for amotivation.

Table B3

Goodness of Fit Statistics for the Revised 9-item Three Factor Model

|  | **df** | **χ²** | **Δdf** | **Δ χ²** | **CFI** | **ΔCFI** | **RMSEA** | **ΔRMSEA** | **SRMR** |
| --- | --- | --- | --- | --- | --- | --- | --- | --- | --- |
| **Initial Sample** | **36** | **47.71** | **-** | **-** | **.95** | **-** | **.06** | **-** | **.07** |
| **Cross Validation Sample** | **24** | **147.38** | **-** | **-** | **.95** | **-** | **.06** | **-** | **.06** |
| Model 1 – Configural Measurement Invariance | 48 | 169.10 | - | - | .94 | - | .07 | - | .06 |
| Model 2 - Metric Measurement Invariance | 54 | 183.14 | 18 | 14.04 | .94 | .00 | .06 | .00 | .07 |
| Model 3 – Scalar Measurement Invariance | 60 | 190.15 | 24 | 21.05 | .94 | .00 | .06 | .00 | .07 |
| Model 4 - Full Uniqueness Measurement Invariance | 69 | 192.48 | 33 | 23.38 | .94 | .00 | .06 | .00 | .07 |

*Note.* A dash indicates that a particular statistic was not applicable for that model. Invariance testing refers to gender invariance.

Table B4

Items, Factor Loadings, and Descriptive Statistics of the 9-item MATSS scores

|  | **Initial Sample** | | | |  | **Cross Validation Sample** | | | |
| --- | --- | --- | --- | --- | --- | --- | --- | --- | --- |
| **Items** | ***M*** | ***SD*** | **λ** | **Θ** |  | ***M*** | ***SD*** | **λ** | **Θ** |
| **Autonomous Motivation** |  |  |  |  |  |  |  |  |  |
| 1. Because I enjoy it | 3.18 | 1.12 | .87 | .24 |  | 3.42 | 1.23 | .81 | .35 |
| 1. Because it is interesting | 2.91 | 1.13 | .76 | .43 |  | 3.11 | 1.23 | .78 | .39 |
| 1. Because the benefits are important to me | 3.36 | 1.16 | .57 | .68 |  | 3.44 | 1.26 | .58 | .67 |
| **Controlled Motivation** |  |  |  |  |  |  |  |  |  |
| 1. Because I feel guilty if I don’t | 2.01 | 0.96 | .36 | .87 |  | 2.11 | 1.14 | .51 | .75 |
| 1. Because other people (e.g., parents, friends) get upset with me if I don’t | 1.97 | 1.13 | .99 | .02 |  | 1.91 | 1.18 | .73 | .47 |
| 1. Because other people (e.g., parents, friends) tell me I should | 2.69 | 1.24 | .51 | .74 |  | 2.42 | 1.32 | .65 | .58 |
| **Amotivation** |  |  |  |  |  |  |  |  |  |
| 1. But I don’t see why I should | 2.19 | 1.01 | .69 | .52 |  | 2.03 | 1.08 | .67 | .55 |
| 1. But I feel it is a waste of time | 2.14 | 1.07 | .79 | .38 |  | 1.91 | 1.09 | .75 | .43 |
| 1. But I don’t see the point | 2.19 | 1.08 | .87 | .25 |  | 1.89 | 1.12 | .81 | .34 |

*Note.* λ = item factor loading; Θ = error term.

**Phase 3: Cross Validation**

**Methods**

The purpose of Phase 3 was test the psychometric properties of the 9-item model derived in Phase 2 using scores obtained from a new sample.

***Participants.*** Of the 1,632 students from the sample described in the main manuscript, 1,283 students (55% male and 45% female) aged 11-15 years (*M* = 12.94, *SD* = 0.54) completed the 9-item MATSS and formed the cross validation sample for purpose of psychometric testing. These participants attended 14 co-educational government high schools in Western Sydney, Australia.

***Measure.*** Students in the cross validation sample completed the 9-item version of the MATSS. This measure included the same definition of active travel, the same stem, and the same 5-point Likert scale as the 16-item MATSS; however, only included the 9 retained items.

***Data Analysis.*** With 1.14% of the data points missing, we again used a maximum likelihood estimator with robust standard errors (MLR). We investigated the factor structure of the same 9-item model examined in Phase 2 using CFA in MPlus (version 7.4) [[24](#_ENREF_24)] with MATSS scores from the new cross validation sample. To test for gender invariance of the parameters within the MATSS model, we carried out a series of multiple group CFA models, as outlined by Muthén and Muthén [[29](#_ENREF_29)], where male and female students formed two separate groups. Finally, interfactor correlations were calculated to assess the nomological network and Raykov’s formula was used to assess composite reliability [[27](#_ENREF_27)].

**Results**

The data showed good fit to the 9-item model: χ² [24] = 147.38, CFI = .95, SRMR = .06, RMSEA = .06 (Table B3). The factor loadings ranged from .58 to .81 for the autonomous motivation subscale, .51 to .73 for the controlled motivation subscale, and .67 to .81 for the amotivation subscale (Table B4). When considering factorial validity across gender, there was evidence of configural, metric, scalar, and full uniqueness invariance as all Δ χ² were non-significant (*p* > .05) and all ΔCFI were <.01 (see Table B3). Among the MATSS subscales, autonomous motivation and amotivation were negatively correlated (*r* = -.40), while controlled motivation and amotivation were positively correlated (*r* = .44), as were autonomous and controlled motivation (*r* = .20). This pattern of correlations suggests nomological validity as the nomological network is in line with the ordered nature of the motivational constructs along the self-determination theory continuum. Subscale reliability results were ρ = .76 for autonomous motivation, ρ = .64 for controlled motivation, and ρ = .79 for amotivation.

**Conclusion**

This preliminary study provided evidence supporting the validity of the MATSS scores among adolescents. These findings enabled the use of the MATSS to investigate whether motivation moderated the relationships between active travel and affective wellbeing.

**Acknowledgements**

We would like to acknowledge the following self-determination theory (SDT) experts who contributed to Phase 1 of this research: Ben Jackson, Cameron Wild, Catherine Sabiston, Cecilie Thogersen-Ntoumani, Elaine Rose, Eleanor Quested, Frédéric Guay, Geoff Williams, Heather Patrick, Ian Taylor, James Dimmock, Jennifer Brunet, Jennifer Laguardia, John Wang, Mark Beauchamp, Marten Vansteenkiste, Martin Hagger, Nikos Ntouamnis, Patrick Graudeau, Pedro Teixeira, Phil Wilson, Richard Ryan, Simon Sebire, Stuart Biddle, and Tao Zhang.

**References**

1. Vallerand RJ, Pelletier LG, Blais MR, Briere NM, Senecal C, Vallieres EF. The Academic Motivation Scale: A measure of intrinsic, extrinsic, and amotivation in education. Educ Psychol Meas. 1992;52(4):1003-17.

2. Glynn SM, Brickman P, Armstrong N, Taasoobshirazi G. Science motivation questionnaire II: Validation with science majors and nonscience majors. Journal of research in science teaching. 2011;48(10):1159-76.

3. Kusurkar R, Croiset G, Kruitwagen C, ten Cate O. Validity evidence for the measurement of the strength of motivation for medical school. Advances in health sciences education. 2011;16(2):183-95.

4. Wang F. Motivation and English achievement: An exploratory and confirmatory factor analysis of a new measure for Chinese students of English learning. North American Journal of Psychology. 2008;10(3):633-46.

5. Markland D, Ingledew DK. The measurement of exercise motives: Factorial validity and invariance across gender of a revised Exercise Motivations Inventory. Br J Health Psychol. 1997;2(4):361-76.

6. Lonsdale C, Hodge K, Rose EA. The Behavioral Regulation in Sport Questionnaire (BRSQ): Instrument development and initial validity evidence. J Sport Exerc Psychol. 2008;30(3):323.

7. Markland D, Tobin V. A modification to the behavioural regulation in exercise questionnaire to include an assessment of amotivation. J Sport Exerc Psychol. 2004;26(2):191-6. doi:10.1123/jsep.26.2.191

8. Goudas M, Biddle S, Fox K. Perceived locus of causality, goal orientations, and perceived competence in school physical education classes. Br J Educ Psychol. 1994;64(3):453-63.

9. McDavid L, Cox AE, Amorose AJ. The relative roles of physical education teachers and parents in adolescents’ leisure-time physical activity motivation and behavior. Psychol Sport Exerc. 2012;13(2):99-107.

10. White RL, Olson R, Parker PD, Astell-Burt T, Lonsdale C. A qualitative investigation of the perceived influence of adolescents’ motivation on relationships between domain-specific physical activity and positive and negative affect. Ment Health Phys Act. 2018.

11. Chan DK, Lonsdale C, Fung HH. Influences of coaches, parents, and peers on the motivational patterns of child and adolescent athletes. Scand J Med Sci Sports. 2012;22(4):558-68.

12. Keegan RJ, Harwood CG, Spray CM, Lavallee DE. A qualitative investigation exploring the motivational climate in early career sports participants: Coach, parent and peer influences on sport motivation. Psychol Sport Exerc. 2009;10(3):361-72.

13. Lubans DR, Lonsdale C, Plotnikoff RC, Smith J, Dally K, Morgan PJ. Development and evaluation of the motivation to limit screen-time questionnaire (MLSQ) for adolescents. Prev Med. 2013;57(5):561-6.

14. Ryan RM, Deci EL. Self-determination theory: Basic psychological needs in motivation, development, and wellness: Guilford Publications; 2017.

15. Marsh HW, Hau K-T, Wen Z. In search of golden rules: Comment on hypothesis-testing approaches to setting cutoff values for fit indexes and dangers in overgeneralizing Hu and Bentler's (1999) findings. Struct Equ Modeling. 2004;11(3):320-41.

16. DeVellis RF. Scale development: Theory and applications. 3rd ed. Los Angeles: Sage Publications; 2012.

17. Dunn JGH, Bouffard M, Rogers WT. Assessing item content-relevance in sport psychology scale-construction research: Issues and recommendations. Meas Phys Educ Exerc Sci. 1999;3(1):15-36.

18. Aiken LR. Three coefficients for analyzing the reliability and validity of ratings. Educ Psychol Meas. 1985;45(1):131-42.

19. Cohen J. Statistical power analysis for the behavioral sciences. New York: Academic Press; 1977.

20. Tabachnick BG, Fidell LS. Using multivariate statistics. 6th ed. Boston: Pearson; 2013.

21. Cohen J. Statistical power analysis for the behavioral sciences. New York: Academic press; 2013.

22. Hardy LL, King L, Espinel P, Cosgrove C, Bauman A. NSW Schools Physical Activity and Nutrition Survey (SPANS) 2010: Full report. Sydney: NSW Ministry of Health.; 2011.

23. Merom D, Tudor-Locke C, Bauman A, Rissel C. Active commuting to school among NSW primary school children: Implications for public health. Health Place. 2006;12(4):678-87.

24. Muthén LK, Muthén BO. Mplus. 7.4 ed. Los Angeles, California: Muthén & Muthén; 2016.

25. Marsh HW, Ellis LA, Parada RH, Richards G, Heubeck BG. A short version of the Self Description Questionnaire II: Operationalizing criteria for short-form evaluation with new applications of confirmatory factor analyses. Psychol Assess. 2005;17(1):81-102.

26. Hu L, Bentler PM. Cutoff criteria for fit indexes in covariance structure analysis: Conventional criteria versus new alternatives. Struct Equ Modeling. 1999;6(1):1-55.

27. Raykov T. Estimation of composite reliability for congeneric measures. Appl Psychol Meas. 1997;21(2):173-84.

28. Cronbach LJ. Coefficient alpha and the internal structure of tests. Psychometrika. 1951;16(3):297-334.

29. Muthén LK, Muthén BO. Mplus statistical analyses with latent variables: User’s guide. 6th ed. Los Angeles, CA: Muthén & Muthén; 2010.
