# Supplementary material for: Domain-specific physical activity and affective wellbeing among adolescents: an observational study of the moderating roles of autonomous and controlled motivation
Source: Int J Behav Nutr Phys Act. 2018 Sep 10;15:87. doi: 10.1186/s12966-018-0722-0 (PMC6131748; doi:10.1186/s12966-018-0722-0)
Supplement: Supplementary file 3 — Figure C1. Self-reported leisure-time physical activity and affect: Structural equation model testing autonomous and controlled motivation as moderators. Figure C2. Objectively measured leisure-time physical activity and affect: Structural equation model testing autonomous and controlled motivation as moderators. Figure C3. Self-reported active travel and affect: Structural equation model testing autonomous and controlled motivation as moderators. Figure C4. Objectively measured active travel and affect: Structural equation model testing autonomous and controlled motivation as moderators. (DOCX 310 kb) [file 12966_2018_722_MOESM3_ESM.docx]

**Appendix C: Figures**

| 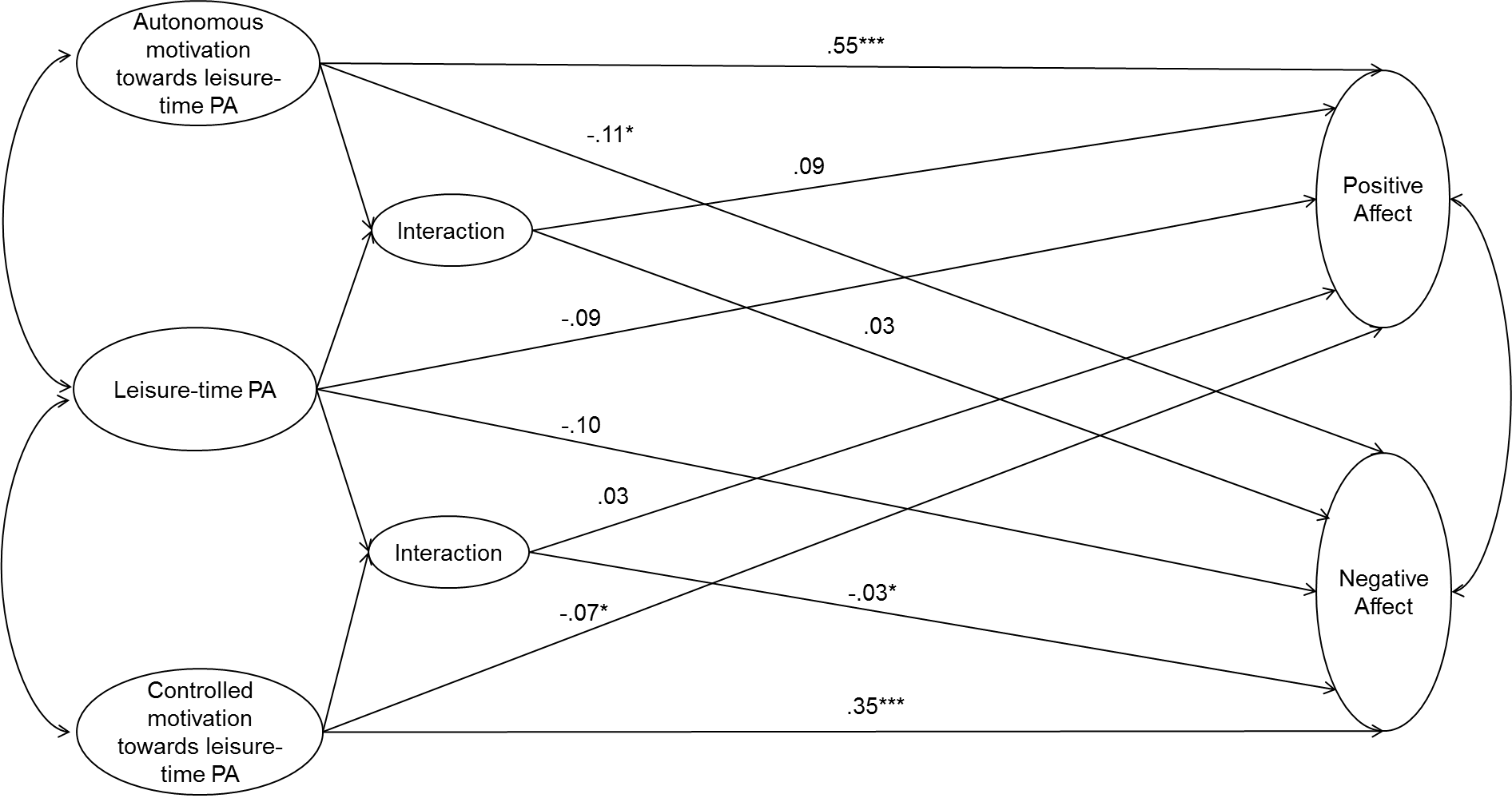 |
| --- |
| *Figure C1.* Self-reported leisure-time physical activity and affect: Structural equation model testing autonomous and controlled motivation as moderators. |
| 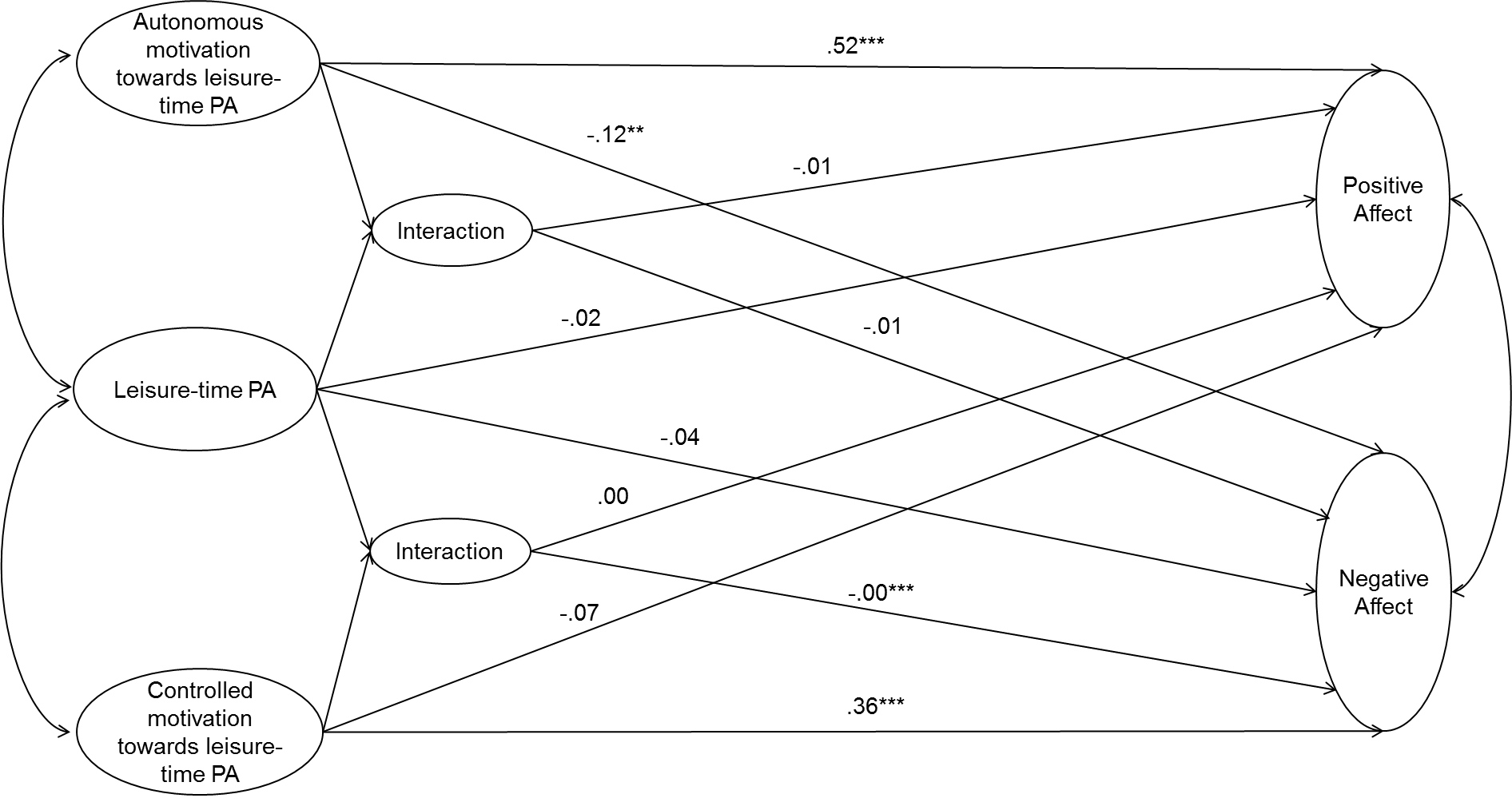 |
| *Figure C2.* Objectively measured leisure-time physical activity and affect: Structural equation model testing autonomous and controlled motivation as moderators. |

| 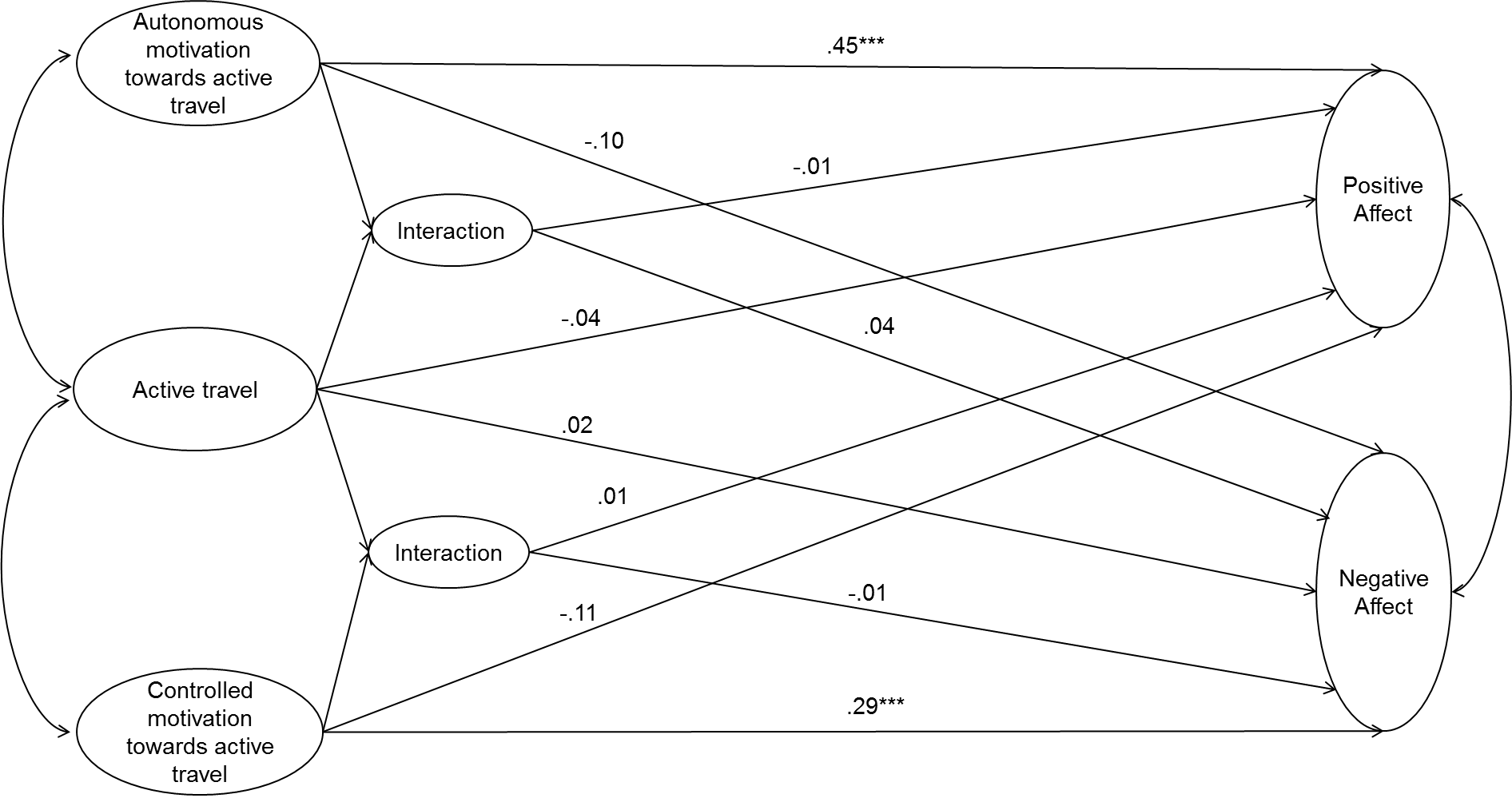 |
| --- |
| *Figure C3.* Self-reported active travel and affect: Structural equation model testing autonomous and controlled motivation as moderators. |
| 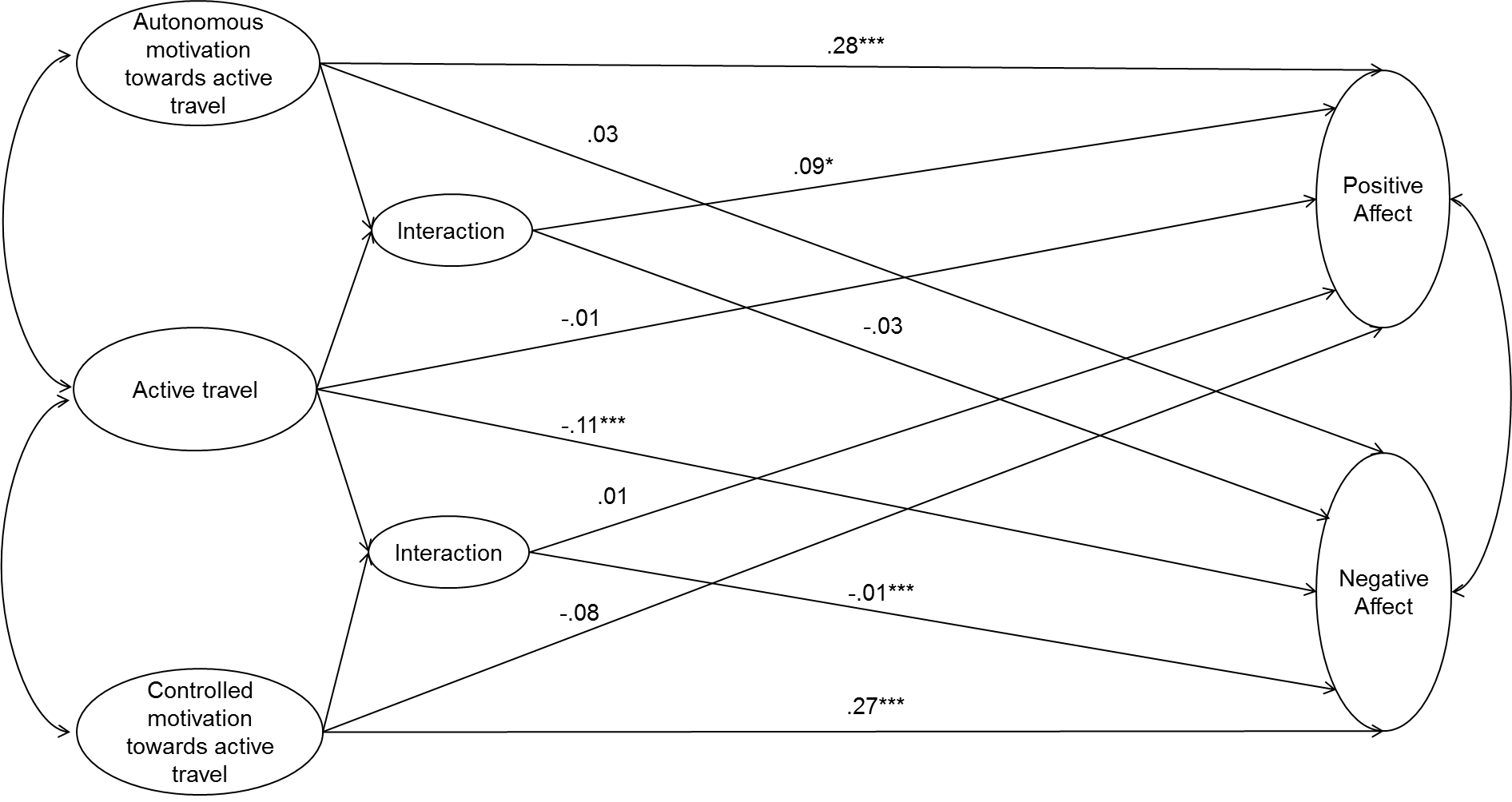 |
| *Figure C4.* Objectively measured active travel and affect: Structural equation model testing autonomous and controlled motivation as moderators. |
