# Supplementary material for: Domain-specific physical activity and affective wellbeing among adolescents: an observational study of the moderating roles of autonomous and controlled motivation
Source: Int J Behav Nutr Phys Act. 2018 Sep 10;15:87. doi: 10.1186/s12966-018-0722-0 (PMC6131748; doi:10.1186/s12966-018-0722-0)
Supplement: Supplementary file 4 — Table D1. Domain-Specific Physical Activity and Affect: Structural Equation Model Testing Autonomous and Controlled Motivation as a Moderators. (DOCX 21 kb) [file 12966_2018_722_MOESM4_ESM.docx]

**Appendix D: Additional Model**

Table D1

*Domain-Specific Physical Activity and Affect: Structural Equation Model Testing Autonomous and Controlled Motivation as a Moderators.*

|  | **Unadjusted model** | | | | |  | | |  | **Adjusted model** | | | | | | | |
| --- | --- | --- | --- | --- | --- | --- | --- | --- | --- | --- | --- | --- | --- | --- | --- | --- | --- |
|  | **β** | ***SE*** | ***p*** | | ***R*²** |  | | |  | **β** | | ***SE*** | | ***p*** | | ***R*²** | |
| **Self-report model** |  |  |  | |  |  | | |  |  | |  | |  | |  | |
| ***Positive Affect*** |  |  |  | |  |  | | |  |  | |  | |  | |  | |
| Leisure-time PA | .04 | .07 | .53 | |  |  | | |  | .01 | | .09 | | .96 | |  | |
| Active travel | .01 | .05 | .84 | |  |  | | |  | .02 | | .06 | | .77 | |  | |
| Autonomous motivation towards leisure-time PA | .44 | .11 | <.001 | |  |  | | |  | .41 | | .11 | | <.001 | |  | |
| Autonomous motivation towards active travel | .35 | .07 | <.001 | |  |  | | |  | .36 | | .09 | | <.001 | |  | |
| Controlled motivation towards leisure-time PA | -.40 | .16 | .01 | |  |  | | |  | -.33 | | .14 | | .02 | |  | |
| Controlled motivation towards active travel | .17 | .13 | .17 | |  |  | | |  | .08 | | .11 | | .49 | |  | |
| Leisure-time PA × Autonomous Motivation | -.03 | .06 | .59 | |  |  | | |  | -.02 | | .07 | | .74 | |  | |
| Active Travel × Autonomous Motivation | -.04 | .06 | .47 | |  |  | | |  | -.04 | | .06 | | .51 | |  | |
| Leisure-time PA × Controlled Motivation | .07 | .05 | .18 | |  |  | | |  | .05 | | .06 | | .41 | |  | |
| Active Travel × Controlled Motivation | -.05 | .05 | .27 | |  |  | | |  | .02 | | .01 | | .14 | |  | |
|  |  |  |  | | .34*** |  | | |  |  | |  | |  | | .36*** | |
| ***Negative Affect*** |  |  |  | |  |  | | |  |  | |  | |  | |  | |
| Leisure-time PA | -.05 | .07 | .51 | |  |  | | |  | -.03 | | .07 | | .68 | |  | |
| Active travel | -.02 | .04 | .63 | |  |  | | |  | -.02 | | .05 | | .66 | |  | |
| Autonomous motivation towards leisure-time PA | -.49 | .09 | <.001 | |  |  | | |  | -.45 | | .11 | | <.001 | |  | |
| Autonomous motivation towards active travel | .04 | .12 | .73 | |  |  | | |  | -.02 | | .16 | | .92 | |  | |
| Controlled motivation towards leisure-time PA | .65 | .18 | <.001 | |  |  | | |  | .59 | | .21 | | <.01 | |  | |
| Controlled motivation towards active travel | -.22 | .16 | .17 | |  |  | | |  | -.09 | | .17 | | .57 | |  | |
| Leisure-time PA × Autonomous Motivation | .07 | .03 | .02 | |  |  | | |  | .05 | | .03 | | .10 | |  | |
| Active Travel × Autonomous Motivation | .03 | .07 | .70 | |  |  | | |  | .07 | | .08 | | .43 | |  | |
| Leisure-time PA × Controlled Motivation | -.06 | .05 | .21 | |  |  | | |  | -.05 | | .06 | | .41 | |  | |
| Active Travel × Controlled Motivation | .11 | .06 | .07 | |  |  | | |  | -.01 | | .02 | | .50 | |  | |
|  |  |  |  | | .26*** |  | | |  |  | |  | |  | | .23*** | |
| **Objective model** |  |  | |  |  | |  |  | | |  | |  | |  | |  |
| ***Positive Affect*** |  |  | |  |  | |  |  | | |  | |  | |  | |  |
| Leisure-time PA | -.01 | .04 | | .77 |  | |  | -.04 | | | .05 | | .36 | |  | |  |
| Active travel | -.03 | .04 | | .36 |  | |  | -.03 | | | .04 | | .47 | |  | |  |
| Autonomous motivation towards leisure-time PA | .44 | .06 | | <.001 |  | |  | .44 | | | .04 | | <.001 | |  | |  |
| Autonomous motivation towards active travel | .06 | .07 | | .41 |  | |  | .07 | | | .08 | | .40 | |  | |  |
| Controlled motivation towards leisure-time PA | -.21 | .14 | | .15 |  | |  | -.21 | | | .14 | | .13 | |  | |  |
| Controlled motivation towards active travel | .15 | .15 | | .33 |  | |  | .13 | | | .13 | | .31 | |  | |  |
| Leisure-time PA × Autonomous Motivation | .01 | .03 | | .71 |  | |  | .00 | | | .03 | | .89 | |  | |  |
| Active Travel × Autonomous Motivation | .08 | .03 | | .01 |  | |  | .08 | | | .04 | | .05 | |  | |  |
| Leisure-time PA × Controlled Motivation | .03 | .07 | | .68 |  | |  | .00 | | | .07 | | .996 | |  | |  |
| Active Travel × Controlled Motivation | -.003 | .04 | | .94 |  | |  | .01 | | | .00 | | .07 | |  | |  |
|  |  |  | |  | .31*** | |  |  | | |  | |  | | .30*** | |  |
| ***Negative Affect*** |  |  | |  |  | |  |  | | |  | |  | |  | |  |
| Leisure-time PA | -.06 | .04 | | .14 |  | |  | -.03 | | | .05 | | .58 | |  | |  |
| Active travel | -.11 | .03 | | <.001 |  | |  | -.10 | | | .03 | | <.001 | |  | |  |
| Autonomous motivation towards leisure-time PA | -.15 | .08 | | .07 |  | |  | -.08 | | | .09 | | .33 | |  | |  |
| Autonomous motivation towards active travel | .07 | .07 | | .33 |  | |  | .07 | | | .09 | | .49 | |  | |  |
| Controlled motivation towards leisure-time PA | .26 | .15 | | .09 |  | |  | .21 | | | .16 | | .19 | |  | |  |
| Controlled motivation towards active travel | .10 | .19 | | .62 |  | |  | .09 | | | .13 | | .47 | |  | |  |
| Leisure-time PA × Autonomous Motivation | -.01 | .04 | | .83 |  | |  | -.04 | | | .04 | | .39 | |  | |  |
| Active Travel × Autonomous Motivation | -.02 | .03 | | .51 |  | |  | -.02 | | | .03 | | .42 | |  | |  |
| Leisure-time PA × Controlled Motivation | .03 | .06 | | .58 |  | |  | .06 | | | .07 | | .39 | |  | |  |
| Active Travel × Controlled Motivation | -.02 | .05 | | .64 |  | |  | -.01 | | | .00 | | <.001 | |  | |  |
|  |  |  | |  | .16*** | |  |  | | |  | |  | | .16*** | |  |

*Note.* PA = physical activity. Adjusted model includes age, sex, socioeconomic status, and body mass index as covariates.

**p* < .05, ***p* < .01, ****p* < .001.
